# Functional Evolution of Avian RIG-I-Like Receptors

**DOI:** 10.3390/genes9090456

**Published:** 2018-09-12

**Authors:** Wanjing Zheng, Yoko Satta

**Affiliations:** Department of Evolutionary Studies of Biosystems, SOKENDAI (The Graduate University for Advanced Studies), Kanagawa 240-0193, Japan; zheng_wanjing@soken.ac.jp

**Keywords:** molecular evolution, natural selection, innate immunity, pattern-recognition receptor, endogenous retrovirus, birds, RIG-I-like receptors (RLRs)

## Abstract

RIG-I-like receptors (retinoic acid-inducible gene-I-like receptors, or RLRs) are family of pattern-recognition receptors for RNA viruses, consisting of three members: retinoic acid-inducible gene I (*RIG-I*), melanoma differentiation-associated gene 5 (*MDA5*) and laboratory of genetics and physiology 2 (*LGP2*). To understand the role of RLRs in bird evolution, we performed molecular evolutionary analyses on the coding genes of avian RLRs using filtered predicted coding sequences from 62 bird species. Among the three RLRs, conservation score and dN/dS (ratio of nonsynonymous substitution rate over synonymous substitution rate) analyses indicate that avian MDA5 has the highest conservation level in the helicase domain but a lower level in the caspase recruitment domains (CARDs) region, which differs from mammals; *LGP2*, as a whole gene, has a lower conservation level than *RIG-I* or *MDA5*. We found evidence of positive selection across all bird lineages in *RIG-I* and *MDA5* but only on the stem lineage of Galliformes in *LGP2*, which could be related to the loss of *RIG-I* in Galliformes. Analyses also suggest that selection relaxation may have occurred in *LGP2* during the middle of bird evolution and the CARDs region of *MDA5* contains many positively selected sites, which might explain its conservation level. Spearman’s correlation test indicates that species-to-ancestor dN/dS of *RIG-I* shows a negative correlation with endogenous retroviral abundance in bird genomes, suggesting the possibility of interaction between immunity and endogenous retroviruses during bird evolution.

## 1. Introduction

The innate immune system is the first-line defense of hosts when encountering infectious pathogens and it is phylogenetically ancient [1]. In the innate immune system, pattern-recognition receptors (PRRs) play a role in pathogen sensing by recognizing evolutionarily conserved molecular structures on pathogens (known as pathogen-associated molecular patterns, PAMPs) [1]. This pathogen recognition triggers the signaling pathways that eventually upregulate the expression of type I interferons, as well as proinflammatory cytokines and chemokines [2,3]. Examples of PRRs include Toll-like receptors (TLRs), NOD-like receptors (NLRs) and RIG-I-like receptors (retinoic acid-inducible gene-I-like receptors, or RLRs).

**Figure 1:**
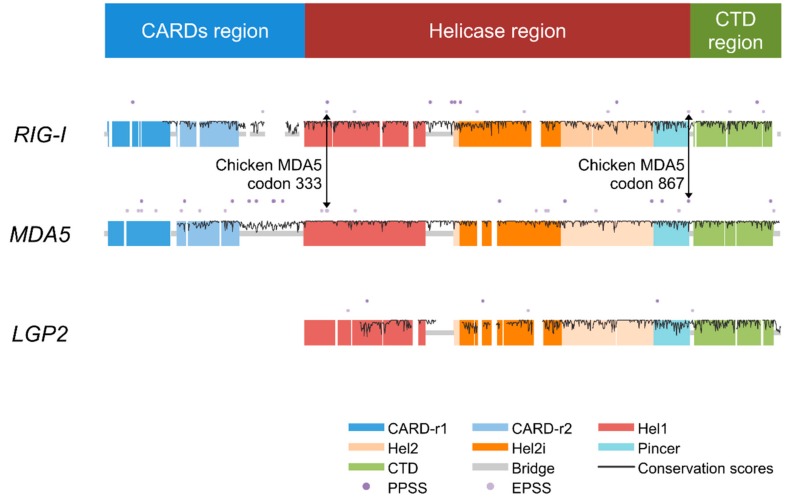
Overview of the three avian RIG-I-like receptors (RLRs). The double-sided black arrows indicate positively selected sites (PSSs) at identical positions in melanoma differentiation-associated gene 5 (*MDA5*) and retinoic acid-inducible gene I (*RIG-I*). The bar at the top indicates the locations of three regions of the RLR genes defined for convenience in this paper. Meanings of dots, colored blocks, and lines in the domain structure sketch of three avian RLRs are denoted in the bottom right of the figure. Conservation scores were calculated only for the sites containing <5% missing data and deletions in total.

The RLRs are a family of three DExD/H box-containing RNA helicases and function as cytoplasmic PRRs sensing non-self RNA [3]. The RLRs are retinoic acid-inducible gene I (*RIG-I*), melanoma differentiation-associated gene 5 (*MDA5*), and laboratory of genetics and physiology 2 (*LGP2*) [4]. RIG-I and MDA5 consist of three functional domains (see the colored blocks at the top of Figure 1): the DExD/H domain (or helicase domain) in the center is responsible for RNA recognition, the two caspase recruitment domains (CARDs) at the N-terminal are responsible for downstream signaling transduction, and the C-terminal domain (CTD) assists in pathogen recognition by binding specific viral RNA [5]. Additionally, a repressor domain (RD) within the CTD is involved in the inhibition of RIG-I signaling in the absence of viruses, while MDA5 does not have an intact RD [5]. MDA5 preferentially recognizes high-molecular-weight double-stranded RNA, while RIG-I preferentially recognizes shorter double-stranded RNA as well as single-stranded RNA [3,4,6]. On the other hand, LGP2 lacks CARDs and therefore does not trigger immune responses but can up- or downregulate the signaling of RIG-I and MDA5 [7,8]. The regulatory function of LGP2 is attributed to its retained helicase domain and RD [9,10].

*RIG-I* may have emerged prior to the appearance of vertebrates, while the other two RLR genes originated in vertebrates [11] by duplication of *RIG-I*. In recent years, the role of avian RLRs on major poultry diseases has been studied; they function as PRRs recognizing RNA viruses in the same manner as mammalian RLRs [12,13,14,15,16]. It was reported that RIG-I expression increased upon avian influenza virus (AIV) infection in ducks [12] and geese (*Anser cygnoides*) [13], and in geese, RIG-I expression also increased upon Newcastle disease virus (NDV) infection [13]. Chickens (*Gallus gallus*) showed weaker resistance to AIV and NDV infection than ducks and geese, and this could be attributed to the loss of RIG-I in chickens [12,14]. In chickens, in which RIG-I has been lost, MDA5 was reported to function as the AIV sensor [15]. Chicken MDA5 was reported to preferentially sense short double-stranded RNA, which is usually done by RIG-I but not MDA5 [16]. Studies also showed that in chicken cells, introduced duck RIG-I could trigger immune responses upon AIV infection [17] and introduced pigeon RIG-I could trigger immune responses upon AIV and infectious bursal disease virus (IBDV) infection [18]. However, studies on the RLRs of other birds are still missing. In addition, the roles of RLRs in the long-term evolution of birds are not fully understood.

Since retroviruses have single-stranded RNA genomes and RIG-I can bind to single-stranded RNA, RIG-I has been considered among the candidate innate sensors of retroviruses [19]. Recently, candidate sensors of retroviruses including RLRs were discussed in relation to their potential influence on endogenous retroviruses (ERVs) [20]. ERVs are sequences within the genome that are highly similar to retroviruses. After infecting a cell, retroviruses integrate into the host’s genome and replicate through host cell machinery [21,22]. If a retrovirus invades and integrates into the germ-line and subsequently becomes transmitted vertically, it becomes an ERV [21]. ERVs can amplify their copy number via retro-transposition or re-infection [23,24,25,26]; and as a result, 8% of the human genome [27] and 11% of the mouse genome [28] consists of ERVs. However, no studies have reported evidence that RIG-I triggers immune responses upon retroviral infection in mammalian cells. RIG-I-dependent pathways were reported to be inhibited in human immunodeficiency virus (HIV)-infected human cells, possibly by HIV proteases [29]. In birds, an equivalent study is still lacking. Interestingly, however, birds have a much smaller amount of ERVs than mammals, ranging from 0.2% to 3.6% of the genome [30]. Thus, it would be interesting to know whether RNA sensors are related to the variance of ERV abundance in hosts since RLRs may function against retrovirus integration during avian evolution.

Here, we report an evolutionary study of avian RLRs using the coding sequences from 62 bird species. We elucidate the evolutionary modes of avian RLRs and examine the evolutionary association of avian RLRs with ERV abundance. Our findings can provide a starting point for future evolutionary studies on the interaction between innate immunity and (endogenous) viruses. This interaction is also an important issue in studies of viral infection and inflammatory diseases. We believe that evolutionary perspectives, especially on organisms that play a role as reservoirs of human disease-causing viruses, such as birds, are informative to a wide range of studies aimed at improving human health.

## 2. Materials and Methods

### 2.1. Sequence Collection and Alignment

Because not all of the 62 bird genomes include RLRs that are fully or correctly annotated, we isolated coding sequences (CDSs) from the genomes of 62 bird species in the NCBI Reference Sequence Database (RefSeq) [31] using BLAST. Exons of *Gallus gallus* Linnaeus, 1758 (chicken) *MDA5* and *LGP2*, and those of *Anser cygnoides* Linnaeus, 1758 (goose) *RIG-I* were used as queries for BLAST searches. For each RLR gene, codon alignment was performed in Molecular Evolutionary Genetics Analysis (MEGA) version 7 [32] (ClustalW) and profile codon alignment was performed in ClustalX version 2.1 [33]. Amino acid sequence alignment of the three avian RLR references was performed using MAFFT (strategy G-INS-I) [34]. In order to ensure the quality of predicted CDSs for analyses, we referred to the scaffolds where BLAST hits were located to manually check the CDS starting, ending and exon-intron boundary regions so as to replace mistaken parts and delete unreliable parts. After this, we concatenated the edited BLAST hits into predicted CDSs.

### 2.2. Six-Class Assessment of Sequence Face Quality

We assigned a predicted CDS into one of six classes from A to F indicating high to low sequence face quality according to the following criteria. Class A: the predicted CDS has both start and termination codons, no premature termination codon (PTC) or frame-shifting insertions and deletions (INDELs) and length ≥90% of the alignment length (including INDELs). Class B: the predicted CDS is not in class A but has a sequence length ≥80% of the alignment length and a total fraction of PTCs and frame-shifting INDELs ≤0.05% of the alignment length. Class C: the predicted CDS is not in the above classes but with a length ≥70% of the alignment length. Class D: the predicted CDS is not in the above classes but with a length ≥50% of the alignment length. Class E: the predicted CDS is not in the above classes but consists of BLAST hits that cover ≥10% with an identity that is ≥70% of at least one query exon. Class F: the rest.

### 2.3. Datasets Preparation

According to the six-class assessment, we grouped the sequences into two datasets: dataset 1 of acceptable data quality (classes A–D; 54 RIG-I, 59 MDA5, and 59 LGP2 sequences) and dataset 2 of good data quality (classes A and B; 39 RIG-I, 57 MDA5, and 31 LGP2 sequences). If an analysis was susceptible to missing data, we applied dataset 2; otherwise, dataset 1 was applied. If an analysis could be susceptible to missing data, dataset 2 was applied; otherwise, dataset 1 was applied. We also generated dataset 3 by combining a subset of dataset 2 with mammal sequences retrieved from GenBank for the bird-mammal comparison. See Table S1 for a detailed list of the datasets.

### 2.4. Molecular Evolutionary Analyses

Conservation scores of amino acid sites were calculated using Scorecons [35] with method valdar01. Average ratio of nonsynonymous substitution rate over synonymous substitution rate (dN/dS) values were estimated with the single likelihood ancestor counting (SLAC) method [36], and then calculated using the Nei–Gojobori [37] method. Gene-wide positive selection was tested using the partitioning approach for robust inference of selection (PARRIS) method [38], a likelihood ratio test (LRT) of the alternative model in which a proportion of sites have evolved under an additional class of dN/dS > 1, against the null model in which the sites have evolved under a class of dN/dS ≤ 1. Episodic positive selection was tested using branch-site random effects likelihood (BSR), an LRT of the alternative model in which a proportion of sites have evolved under dN/dS > 1 on a specific branch (Figure S1) [39]. The statistical significance was determined by the *p*-value corrected with the Holm–Bonferroni method [40] of the LRT using the asymptotic distribution of a mixture of two χ^2^ distributions [39] and a corrected *p*-value < 0.05 was considered statistically significant.

Positively selected sites (PSSs) were detected using SLAC [36] and the mixed effects model of evolution (MEME) [41]. SLAC first reconstructs ancestral codons with maximum likelihood, and based on this reconstruction, the proportion of nonsynonymous substitutions to all substitutions is tested at each codon against the mean value. A significant excess to the mean value would indicate positive selection at that site. Statistical significance of the excess is determined by the *p*-value using an extended binomial distribution [36]. In contrast, MEME applies a branch-site random effects phylogenetic framework that allows the distribution of dN/dS to vary from site to site as well as from branch to branch, which allows MEME to identify instances of both episodic and pervasive positive selection. Among the parameters of MEME, there is a category containing an unrestricted dN parameter for an alternative model, while the null model has the dN parameter of this category restricted to being ≤ dS. Statistical significance of the alternative model at a site will indicate positive selection, and the significance is determined by the *p*-value of the LRT using the asymptotic distribution of a mixture of three χ^2^ distributions [41]. For both methods, a *p*-value < 0.05 can be basically considered as statistically significant evidence of PSSs. Further, we made an integrative determination of PSSs based on this basic criterion. Results from MEME analyses (*p* < 0.05) covers all the PSSs detected by SLAC (*p* < 0.05); thus, we categorized these PSSs detected by both methods as pervasive positively selected sites (PPSSs). We simultaneously categorized the sites detected with *p* < 0.01 in MEME and *p* ∈ (0.05, 0.1) in SLAC as episodic positively selected sites (EPSSs). The criterion of EPSS was cautiously set due to the lack of alternative methods with sufficient power equivalent to MEME to detect EPSSs. To detect the PSSs of birds, dataset 2 was used.

RELAX [42] was used to detect changes of selection intensity. RELAX assumes that positive and/or negative (purifying) selection, if it exists, would be under the same relaxation or intensification. We denote positive and/or negative selection as ‘selection’ for short in this paper. In RELAX, the intensity of selection of two appointed groups of branches in a phylogenetic tree is compared. The result of the comparison is expressed with an optimized parameter K, whereby K > 1 indicates an intensified and K < 1 indicates a relaxed selection of the test group relative to the reference group, as is in the alternative model, while the null model shows K = 1. Statistical significance of the alternative model is determined by the *p*-value of the LRT using standard χ^2^ asymptotic distribution [42] and a *p*-value < 0.05 is considered statistically significant. Fifty million years ago (MYA) were chosen as the boundary of old and young branch groups because it is located approximately in the middle of the estimated time of ~102 MYA, tracing back to the ancestor of extant birds [43]. If a branch ended no later than the boundary, it was assigned to the old branch group (test group); otherwise, it was assigned to the young branch group (reference group). For RELAX analysis of the comparison between birds and mammals, we made a joint alignment of bird and mammal CDSs for each of the three RLRs.

The necessary phylogenetic information of the 62 birds for analyses were comprised of the phylogeny reconstructed from 48 birds with whole-genome data [43], the suggested topological positions by BirdTree [44] and the suggested divergence times by TIMETREE [45] for other species. The phylogenetic information of 10 mammals was also cited from a reported phylogeny reconstruction with whole-genome data [46]. PARRIS, BSR, SLAC, MEME and RELAX analyses were performed with HyPhy [47] on the webserver Datamonkey [48]. Protein structure images were processed and exported using Chimera [49].

### 2.5. Correlation Analysis of Species-to-Ancestor dN/dS of Avian RLRs vs. ERV Abundance

Spearman’s correlation tests were performed on species-to-ancestor dN/dS versus ERV abundance and a *p*-value < 0.05 was considered statistically significant. The dataset comprised the species in dataset 1 that intersected with the 48 species [43] with phylogenetic relationships reconstructed using whole-genome sequences (Table S1). Here, species-to-ancestor dN/dS was used as an index of long-term average functional constraint. The ancestral sequences for calculating the species-to-ancestor dN/dS were inferred from dataset 1 with the maximum likelihood method in MEGA 7 [32], and *Alligator mississippiensis* Daudin, 1802 (American alligator) was used as an outgroup. ERV abundance was defined as the ERV copy number divided by the genome size (Gb) of a species and was calculated based on published data [30]. Since the significance may be biased if two biological traits were not taken independently from a common distribution but from a branching phylogeny, the data used in correlation tests had been transformed into normalized phylogenetic independent contrasts (PICs) [50] using DendroPy [51] with known tree topology and branch lengths.

## 3. Results

### 3.1. Face Quality of Predicted CDSs

Since genome annotations are not completely satisfactory, predicted CDSs were isolated from the genome data of 62 bird species (Table S1) using BLAST. Subsequently, manual procedures were applied to optimize the quality of data for evolutionary analyses (see Materials and Methods for details).

We assessed the face quality of each predicted CDS, namely, the extent of completeness as a practical CDS used in the analysis. According to a series of subjective criteria (see Materials and Methods for details), we assigned each predicted CDS to one of the six classes (A–F; high to low face quality). *MDA5* had the largest number of cases in which a species has a predicted CDS of good face quality in class A or B (*n* = 57) and *LGP2* had the smallest (*n* = 31), while *RIG-I* had the largest number of cases of the worst class F (*n* = 8) and *LGP2* had the largest number of cases of the second worst class E (*n* = 12). The six-class assessment also showed variety among species (Figure S1). Even though the *Meleagris gallopavo* Linnaeus, 1758 (turkey), chicken, *Coturnix japonica* Temminck and Schlegel, 1849 (Japanese quail) and *Chaetura pelagica* Linnaeus, 1758 (chimney swift) had finely assembled genomes, their *RIG-I* was assigned to class F, which suggest putative loss of *RIG-I*.

### 3.2. Gene-Wide Conservation and dN/dS Levels of Avian RLRs

To evaluate the conserved mode of evolution of the three avian RLRs, average conservation scores and average dN/dS ratios were calculated.

We first calculated the conservation scores of amino acid sites of the three RLRs (dataset 2). Between the two signaling receptors, MDA5 (0.933 ± 0.096, 43%) showed a slightly higher average conservation score and proportion of invariant sites than those of RIG-I (0.913 ± 0.114, 37%) (Table 1). We then excluded the CARDs region (defined as the two CARDs with in-between or flanking nondomain regions in this study), such that the scores became comparable with LGP2, and found that MDA5 (0.953 ± 0.080, 50%) showed a much higher average conservation score and proportion of invariant sites than RIG-I (0.915 ± 0.112, 37%) and LGP2 (0.881 ± 0.139, 36%). Notably, MDA5 had a lower conservation level than RIG-I in CARDs; therefore, the exclusion of the CARDs resulted in a further lifted average conservation score for MDA5 (Table 1). When we analyzed the helicase region only (helicase domain with the pincer in this study), this region contributed the most to the leading conservation level of MDA5 over the other two. For both the helicase and CTD regions (here, this refers to the C-terminal domain and its flanking nondomain regions), LGP2 showed a slightly lower conservation level than RIG-I and MDA5 (Table 1). Each of the three avian RLRs had an average conservation score of nearly or over 0.9 and a proportion of invariant sites of over 30%, which indicates an overall high conservation level in the three avian RLRs. Conservation scores across sites are indicated in Figure 1.

We checked dataset 2 for the conservation level of two ubiquitinated sites in duck CARDs, Lys167 and Lys193 (duck site number) [52]. Polyubiquitin chains that are attached to lysine residues by the ubiquitin ligase TRIM25 are necessary for CARDs activation in humans but seem unnecessary in ducks; instead, noncovalent ubiquitin chains may play more important roles [52]. We found that Lys167 is invariant among the alignment of dataset 2, while Lys193 shows substitutions in the two crow species (Thr) and the carmine bee-eater (Glu).

Next, we estimated the average dN/dS ratio of the three avian RLRs (dataset 2) with two methods: the SLAC [36] and Nei–Gojobori methods [37]. With full sequences of bird RLRs, *MDA5* shows a similar dN/dS to that of *RIG-I*, with their 95% confidence intervals (CI) based on the SLAC method largely overlapping (Table 2). This result shows consistency with that of a smaller dataset (dataset 3, see Figure S2 and Materials and Methods). We looked into dataset 3 for some more details. When the CARDs region is excluded, dN/dS of *RIG-I* does not change much, while that of *MDA5* decreases and becomes lower than that of *RIG-I* or *LGP2*. This tendency is consistent with the observation of conservation scores. The dN/dS values of all three avian RLRs are higher than the average level of avian protein coding genes (Figure S3) [53]. However, the dN/dS values of the RLRs are much smaller than 1, which is supported by the 95% CIs of SLAC (Table 2). This indicates that purifying selection is still the dominant mode of selection acting on the three avian RLRs.

Conservation score and dN/dS results together suggest that for avian MDA5, the CARDs region might have experienced weaker functional constraint or stronger positive selection while the helicase and CTD regions may have experienced stronger functional constraint or weaker positive selection compared with the other two RLRs. Conservation score results suggest that avian LGP2 may have faced slightly weaker functional constraint or slightly stronger positive selection compared with the helicase-CTD regions of the other RLRs.

From the comparison of contrasting patterns of dN/dS between birds and mammals without the CARDs region, we found that birds and mammals share similar dN/dS levels for each of the three RLRs (dataset 3, Table 2). However, different from the pattern observed in birds, with the CARDs region, dN/dS values of *RIG-I* and *MDA5* in mammals both increase and thus *RIG-I* still has higher dN/dS than *MDA5* (reported mammal data [54] and dataset 3). Such contrasting patterns are consistent between the results of the two methods (SLAC and Nei–Gojobori) and are supported by the 95% CI of SLAC.

### 3.3. Positive Selection and Positively Selected Sites in Avian RLRs

To evaluate the extent of adaptive evolution, we examined gene-wide positive selection. Using a *p*-value of 0.05 as the significance level, positive selection was detected in *RIG-I* (*p* = 1.6 × 10^−4^) and *MDA5* (*p* = 4.7 × 10^−10^) but not in *LGP2* (*p* = 0.999). However, episodic (lineage-specific) positive selection (corrected *p* = 0.014) was detected in *LGP2* in the stem lineage of the Galliformes (the ancestral branch of the chicken, Japanese quail, and turkey) (Figure S4).

**Figure 2:**
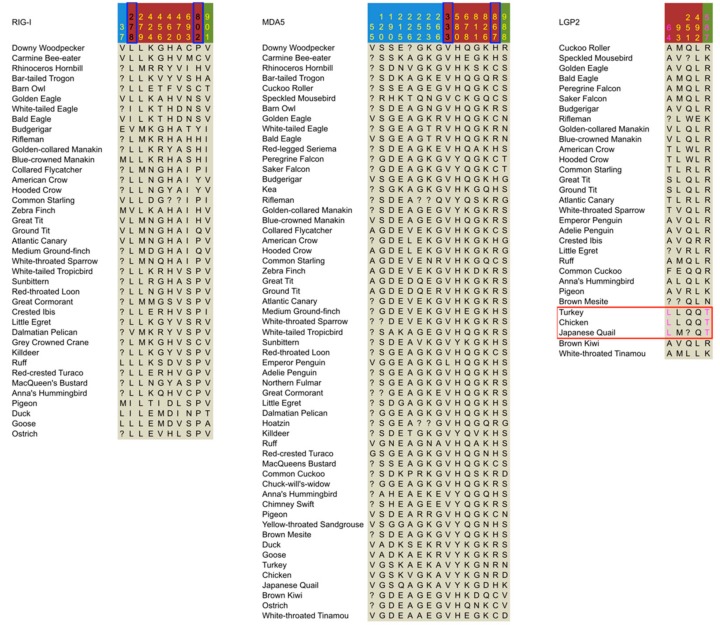
Summary of positively selected sites identified in the three avian RIG-I-like receptors. Colors of site numbers indicate the type of positively selected sites (PSSs): yellow corresponds to pervasive positively selected sites (PPSSs), magenta corresponds to the episodic positively selected sites (EPSSs) showing specific substitutions in the LGP2 of Galliformes, and black corresponds to other EPSSs. The LGP2 of Galliformes is indicated by a red rectangular frame. The blue frames on the site numbers indicate PSSs located identically in RIG-I and MDA5. Site numbers are according to chicken sequences for MDA5 and LGP2, and the goose sequence for RIG-I. Background colors of site numbers represent coding sequence (CDS) regions defined for convenience in this paper: sky blue for the caspase recruitment domains (CARDs) region, dark red for the helicase region, and green for the C-terminal domain (CTD) region.

To determine the sites that are vital for adaptive evolution, we performed positively selected sites (PSSs) detection of two categories, PPSSs and EPSSs (see Materials and Methods), based on the results of two distinct detecting methods, SLAC and MEME (Table 3) [41]. *MDA5* has the highest number and density of PPSSs and EPSSs, while *LGP2* has the lowest (Figure 1 and Figure 2). This again suggests that the level of positive selection acting on *LGP2* is lower than the other RLRs, which cannot explain the lower conservation level of *LGP2* compared with the other two. When comparing *MDA5* and *RIG-I*, the density of PSSs (number of PSSs over total number of sites, expressed as %) differed the most in the CARDs region: *MDA5* had 15 PSSs (5.05% of the region), while *RIG-I* had two PSSs (0.82% of the region), differences which were not marked in other regions (Figure 1). This suggests that positive selection may be the cause of the lower conservation level of the *MDA5* CARDs region rather than that of *RIG-I.*

We further examined EPSSs showing specific amino acid substitutions in the *LGP2* of Galliformes, since *LGP2* displayed episodic positive selection in the stem lineage of the Galliformes, and such EPSSs can be important for the functional adaptation of LGP2 in Galliformes. We found two EPSSs showing specific substitutions in Galliforme LGP2, with one substitution (Ala64Leu, chicken site number used) located in the CARDs region and the other (Arg587Thr) in the helicase region (Figure 2). Among all the substitutions on either of the two sites in the alignment, the amino acid replacement that could lead to the Galliforme-specific substitution has a relatively small rate in the Dayhoff matrix [55]. Particularly for Arg587Thr in Galliformes, not only is the corresponding amino acid replacement rate much lower than other substitutions occurring at this site, but also the chemical nature is uniquely changed from acidic to neutral nonpolar. Since chicken Thr587 is located in the 3′ end-binding loop [56], this substitution may have changed the double strain RNA (dsRNA) end-binding function of LGP2 in Galliformes.

**Figure 3:**
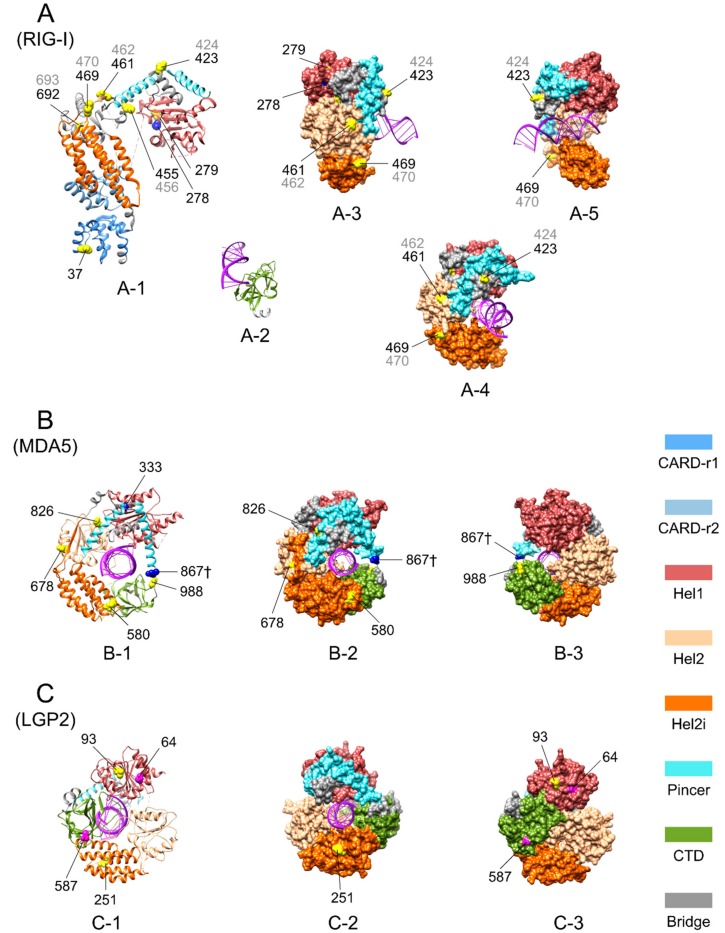
Three-dimensional (3D) structures of avian RIG-I-like receptors with some positively selected sites (PSSs) marked. Pervasive positively selected sites (PPSSs), the episodic positively selected sites (EPSSs) with specific substitutions in LGP2 of Galliformes, and the PSSs located identically in MDA5 and RIG-I are mapped on available 3D protein structure images of duck RIG-I, partial chicken MDA5, and LGP2 (the MDA5 image does not include the caspase recruitment domains (CARDs) region; some PSSs are within the missing regions of the images and thus cannot be shown). (**A**) Duck RIG-I. Original image PDB IDs: 4a2w for A-1, 4a2x for A-2 and 4a36 for A-3, A-4 and A-5. In (**A**), A-1 shows the ribbon diagram of domains except the C-terminal domain (CTD) and A-2 shows the CTD; A-3 to A-5 show surface displays of the helicase domain from various angles. (**B**) Chicken MDA5. Original image PDB ID: 5JCH. (**C**) Chicken LGP2. Original image PDB ID: 5JBJ. In (**B**,**C**), from left to right, the ribbon diagram, surface display diagram and the surface display diagram rotated 180° on the vertical axis are shown. Colors of domains are denoted in the bottom right of the figure. Sites marked in yellow or with † appended to the site number represent PPSSs; sites in magenta represent EPSSs with specific substitutions in LGP2 of Galliformes; sites in blue represent PSSs located identically in RIG-I and MDA5. Marked sites are shown with the sphere display of side chains for the purpose of highlighting. Site numbers pointed to marked sites correspond to the chicken sequence for MDA5 and LGP2, to the duck sequence in black, and to the goose sequence in grey if different from that of duck for RIG-I.

Three-dimensional images of duck RIG-I [57], chicken MDA5 and LGP2 with bound RNA [56] showed that almost all of the PPSSs and abovementioned EPSSs are exposed on the surface of the proteins, and none of them are located within close contact to the bound RNA (Figure 3). This suggests that these PPSSs and EPSSs may play a role in minor adjustments to the process of pathogen recognition and CTD regulation rather than direct changes to the core function of pathogen binding.

Other PSSs may also have interesting functional effects. Two PSSs are located identically in both MDA5 and RIG-I (Figure 1 and Figure 2) and are located separately at each of the two tails of the helicase region. Additionally, the only PSS (a PPSS) in RIG-I CARDs is located within the spliced-out sequence (namely the skipped exon 2 that encodes part of the first CARD) of a splice variant observed in duck [52]. This splice variant also exists in mammalian RIG-I [58] and may also exist in other birds. Since the incomplete CARDs of the splice variant cannot interact with TRIM25, its CARDs cannot be ubiquitinated covalently or noncovalently and thus are not activated [52]. The reason why these sites experienced positive selection needs future investigation.

### 3.4. Changes of Selection Intensity in Avian RLR Evolution

To examine whether relaxation of functional constraint occurred and contributed to the contrasting patterns of conservation level among the three RLRs in birds, we tested selection intensity change using RELAX [42]. RELAX compares the selection (positive and/or negative, see Materials and Methods) intensity between two groups of branches in a phylogeny. In our study, each RELAX analysis was performed independently in each of the RLRs.

First we considered the possibility that selection relaxation on *LGP2* or *RIG-I* started in the ancestor of birds, by comparing the selection intensity between the groups of birds and mammals of dataset 3. If selection relaxation were detected in the bird group compared with the mammal group, it would suggest that selection relaxation began in the ancestor of birds. The results show that no significant (0.05 level) difference of selection intensity was detected on *LGP2* or *RIG-I*, suggesting that selection relaxation did not occur in the ancestor of birds and thus is not a contributor to the lower conservation level of *LGP2* or *RIG-I* relative to *MDA5*.

We then examined whether relaxation could occur during the middle of bird evolution on *LGP2* or *RIG-I* by comparing the intensity of selection between old and young branches of *LGP2* and *RIG-I* in dataset 2. We divided the branches into old branch (test) group and young branch (reference) group. The old branch group represents the earlier stages of bird evolution, while young branch group represents the later stages. When all regions of each gene were used, selection relaxation on the young branch group compared with the old branch group was detected on *LGP2* (K = 0.82, *p* = 0.042) but not on *RIG-I*. This supports the notion that selection relaxation on *LGP2* might have occurred in the middle of bird evolution and contributed to the lower conservation level of *LGP2* relative to *MDA5*.

Furthermore, we examined whether the CARDs region of *MDA5* has experienced selection relaxation during bird evolution. We compared selection intensity on the *MDA5* CARDs region between birds and mammals, as well as between the old and young branches. No significant (0.05 level) relaxation was detected in either test, suggesting that selection relaxation at the beginning or middle of bird evolution did not occur and thus could not contribute to the lower conservation level of the CARDs region of avian *MDA5*.

### 3.5. Correlation between Functional Constraint of Avian RLR Genes and ERV Abundance

**Figure 4:**
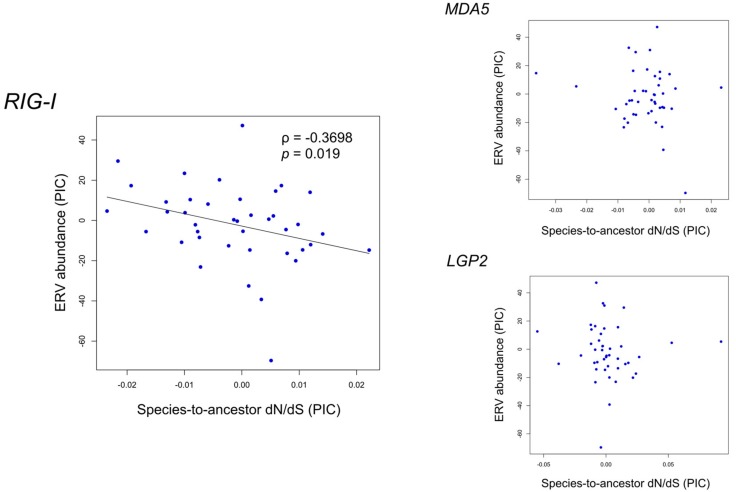
Relationship between the phylogenetic independent contrasts (PICs) of endogenous retroviruses (ERV) abundance and species-to-ancestor dN/dS of three avian RIG-I-like receptors. When Spearman’s correlation has a *p* < 0.05, a trend line is shown. Note that the slope is not equal to Spearman’s rank correlation coefficient (‘ρ’ in the figure).

The small ERV abundance of birds (compared with mammals) and the RNA sensing ability of RLRs raised our query about whether an association exists between the functional constraint of avian RLR genes and ERV abundance during bird evolution. To address this, we performed Spearman’s correlation test on species-to-ancestor dN/dS of avian RLRs versus ERV abundance. ERV information was retrieved from a reported study in which endogenous viral elements including ERVs in 48 birds were mined using a library of representative viral protein sequences derived from a known species list [30]. To avoid effects of phylogenetic relationships among samples, phylogenetic independent contrasts (PICs) of species-to-ancestor dN/dS of avian RLRs and ERV abundance were used in the correlation tests. With a *p*-value of 0.05 as the significance level, a negative correlation was found between the ERV abundance and the species-to-ancestor dN/dS of *RIG-I* (ρ = −0.3698, *p* = 0.019) (Figure 4) but not *MDA5* or *LGP2*. Since low dN/dS values indicate high functional constraint, this result suggests that a positive association may exist between ERV abundance and functional constraint on *RIG-I* during avian evolution.

Furthermore, we investigated the location of the nodes with a difference larger than 30 or 20 of the ranks between species-to-ancestor dN/dS and ERV abundance out of the 40 nodes in the tree (Figure 4). We found that these nodes are located widely across the tree without gathering in specific clades (Figure S5), indicating that the correlation is contributed from various bird lineages and reflects a pervasive evolutionary mode in birds.

## 4. Discussion

The conservation and the dN/dS levels identified in our study suggest that purifying selection is the major driving force in the evolution of avian RLRs. However, evolutionary modes differ among the three avian RLR genes. Avian *MDA5* may be more functionally conserved and may have been under the strongest purifying selection among the RLRs, since *MDA5* shows the lowest level of dN/dS and its encoded amino acid sequences show the highest level of conservation, especially in the helicase-CTD region. *MDA5* may have also been under the strongest positive selection among the three, since: (1) it has the highest number and density of PSSs and (2) it exhibits a higher PSS density but not selection relaxation, which may explain the lower conservation level in its CARDs region. The above suggestions imply that *MDA5* may not only bear constant functional importance but may also be a hotspot of genetic adaptation in immune pathways during bird evolution. The exceeded PSS density of *MDA5* over *RIG-I* in the CARDs region (especially the nondomain region following CARDs) suggests that signal transduction behavior can be an important aspect in MDA5-related adaptation apart from pathogen recognition.

On the other hand, avian *RIG-I* seems to have undergone a lesser degree of natural selection than *MDA5*. However, its evolution has unique characteristics. Since the *RIG-I* sequences of the three Galliformes in our datasets (turkey, chicken, Japanese quail) and the chimney swift were from high-quality genome assembles but were assigned to the lowest face quality class F, *RIG-I* loss is suggested to have occurred in these species. Since *RIG-I* loss has been reported in chickens [12], it would be interesting to also confirm *RIG-I* loss in turkeys and Japanese quails. If confirmed, *RIG-I* loss might have occurred in the stem lineage of Galliformes. The *RIG-I* loss in chimney swifts also needs to be confirmed. If those losses were confirmed, an interesting question would be raised: whether or not those losses of genes were adaptive, and if not, how the losses were compensated. More interestingly, a positive association was identified between the functional constraint on *RIG-I* and ERV abundance. This association suggests a certain interaction between *RIG-I* and ERVs during bird evolution. For example, *RIG-I* might respond to expressed ERVs. This possibility is worthy of further study since previous studies in mammals showed that RIG-I is the only RLR that can detect single-stranded RNA [3,4,6], which is the exact component of transcribed ERVs as well as genomes of retroviruses. RIG-I was also reported to have induced immune responses against the introduced genome of the retrovirus HIV in human cells [29]. Since knowledge of the innate immunity against retroviruses/ERVs is still limited, our preliminary study here can inform future studies on innate immunity against retroviruses and the evolutionary role of ERVs in vertebrates.

As for avian *LGP2*, we found a heterogeneous evolutionary mode, including episodic positive selection on *LGP2* in the stem lineage of Galliformes. This could be related to the putative loss of *RIG-I* in the Galliforme ancestor as mentioned above. Since MDA5 does not have an intact RD and can be regulated by the RD of LGP2, loss of *RIG-I* might change the regulatory manner of LGP2 on MDA5 through evolution with a footprint of positive selection. In addition, selection (positive and/or negative) intensity might have intensified in the origin of birds and relaxed in the middle of bird evolution. This relaxation might have led to the relatively low conservation level of *LGP2* among the three RLRs.

We also found evidence supporting the idea that evolutionary modes of RLRs may partially differ between birds and mammals. In mammals, *RIG-I* has higher dN/dS than *MDA5*; when the CARDs region was excluded, all dN/dS values decrease but such contrasting patterns remain. However, in birds, *RIG-I* has a similar dN/dS to that of *MDA5* and only the dN/dS of *MDA5* decreases after excluding the CARDs region (that is, the dN/dS of *MDA5* becomes lower than that of *RIG-I*). The difference between birds and mammals could be related to the differences in the evolutionary modes among gene regions and the differences between birds and mammals in the functional balance among the three RLRs during evolution.

This study reports the first evidence of an evolutionary association between RIG-I and ERV abundance. Future work is needed to investigate whether such an association also occurs in mammals and other vertebrates. The expansion of ERVs is one of the characteristics of mammalian genome evolution and is related to the complicated evolutionary interaction between host and ERVs/retroviruses [20]. Although most ERVs are defective due to mutations, some of them retain the capacity for expression and even infection. Moreover, the expressed ERVs could be related to numerous inflammatory diseases [19,20]. On the other hand, ERVs are sometimes co-opted by the host and become an important source of functional or regulatory innovation, as is known in mammalian evolution [20,59]. Regarding the role of the immune system in the evolution of ERV abundance, a number of questions could be asked: Can evolutionary interactions between hosts and ERVs/retroviruses restrict the innate immune mechanisms against retroviruses? Could evolutionary interactions between hosts and ERVs/retroviruses in birds be less complex than that in mammals since avian genomes contain a smaller proportion of ERVs? Do some innate immune sensors and pathways function more effectively against ERVs/retroviruses in birds than in mammals as a consequence? We believe that the further exploration of molecular evolutionary signals is one of the important approaches to answering such questions.

## Figures and Tables

**Table 1:** Conservation scores of the three avian RIG-I-like receptors (retinoic acid-inducible gene-I-like receptors, or RLRs).

Domain Regions	RIG-I	MDA5	LGP2
Three regions			
Avg. ± S.D.	0.913 ± 0.114	0.933 ± 0.096	N/A
Invariant/all	37%	43%	
Without CARDs region			
Avg. ± S.D.	0.915 ± 0.112	0.953 ± 0.080	0.881 ± 0.139
Invariant/all	37%	50%	36%
CARDs region			
Avg. ± S.D.	0.903 ± 0.121	0.861 ± 0.113	N/A
Invariant/all	35%	15%	
Helicase region			
Avg. ± S.D.	0.914 ± 0.111	0.955 ± 0.079	0.883 ± 0.138
Invariant/all	36%	53%	35%
CTD region			
Avg. ± S.D.	0.916 ± 0.116	0.942 ± 0.085	0.877 ± 0.141
Invariant/all	41%	39%	39%

RIG-I: retinoic acid-inducible gene I; MDA5: melanoma differentiation-associated gene 5; LGP2: laboratory of genetics and physiology 2. SD: standard deviation; CARDs: caspase recruitment domains; CTD: C-terminal domain.

**Table 2:** Mean dN/dS of RLR genes in birds and mammals.

Domain Regions and Datasets		No. of Species		dN/dS (95% Confidence Interval)		
		*RIG-I/MDA5/LGP2*		*RIG-I*	*MDA5*	*LGP2*
Dataset 2 and literature						
All regions	Birds (dataset 2)	39/57/31	†	0.385	0.369	0.222
				(0.368, 0.401)	(0.355, 0.383)	(0.211, 0.234)
	Mammals [54]	42/46/46	‡	0.403	0.293	0.221
				(0.390, 0.416)	(0.284, 0.302)	(0.213, 0.230)
Dataset 3						
Three regions	Birds	9/12/7	†	0.350	0.384	—
				(0.325, 0.376)	(0.357, 0.411)	
			‡	0.237	0.237	—
	Mammals	7/10/8	†	0.408	0.330	—
				(0.374, 0.443)	(0.306, 0.356)	
			‡	0.283	0.225	—
Without CARDs region	Birds	9/12/7	†	0.352	0.284	0.200
				(0.323, 0.383)	(0.257, 0.312)	(0.180, 0.220)
			‡	0.237	0.185	0.253
	Mammals	7/10/8	†	0.370	0.260	0.240
				(0.334, 0.410)	(0.234, 0.288)	(0.219, 0.264)
			‡	0.262	0.180	0.254

†: SLAC method. ‡: Nei–Gojobori method.

**Table 3:** Number of positively selected sites identified in the three avian RLR genes.

Sites	No. Codons (Proportion to the Alignment)					
	*RIG-I*		*MDA5*		*LGP2*	
Alignment	933	(100%)	1025	(100%)	677	(100%)
PPSS	8	(0.9%)	14	(1.4%)	3	(0.4%)
EPSS	10	(1.1%)	17	(1.7%)	3	(0.4%)
Total	18	(1.9%)	31	(3.0%)	6	(0.9%)

PPSS: pervasive positively selected sites; EPSS: episodic positively selected sites.

## Supplementary Materials

The following are available online at http://www.mdpi.com/2073-4425/9/9/456/s1, Table S1: Datasets of RLR CDSs, Figure S1: Six-class face quality assessment of the predicted avian RLR CDSs, Figure S2: Phylogeny of the species used for comparative analyses of RLRs between birds and mammals (dataset 3), Figure S3: dN/dS of the three avian RLRs compared with average level of coding genes in birds, Figure S4: BSR analysis of avian LGP2.
