# Supplementary material for: Functional Evolution of Avian RIG-I-Like Receptors
Source: Genes (Basel). 2018 Sep 12;9(9):456. doi: 10.3390/genes9090456 (PMC6162795; doi:10.3390/genes9090456)
Supplement: Supplementary file 1 [file genes-09-00456-s001.zip › genes-349720-Supplementary figures-revised.docx]

Supplementary Figures

Functional Evolution of Avian RIG-I-like Receptors


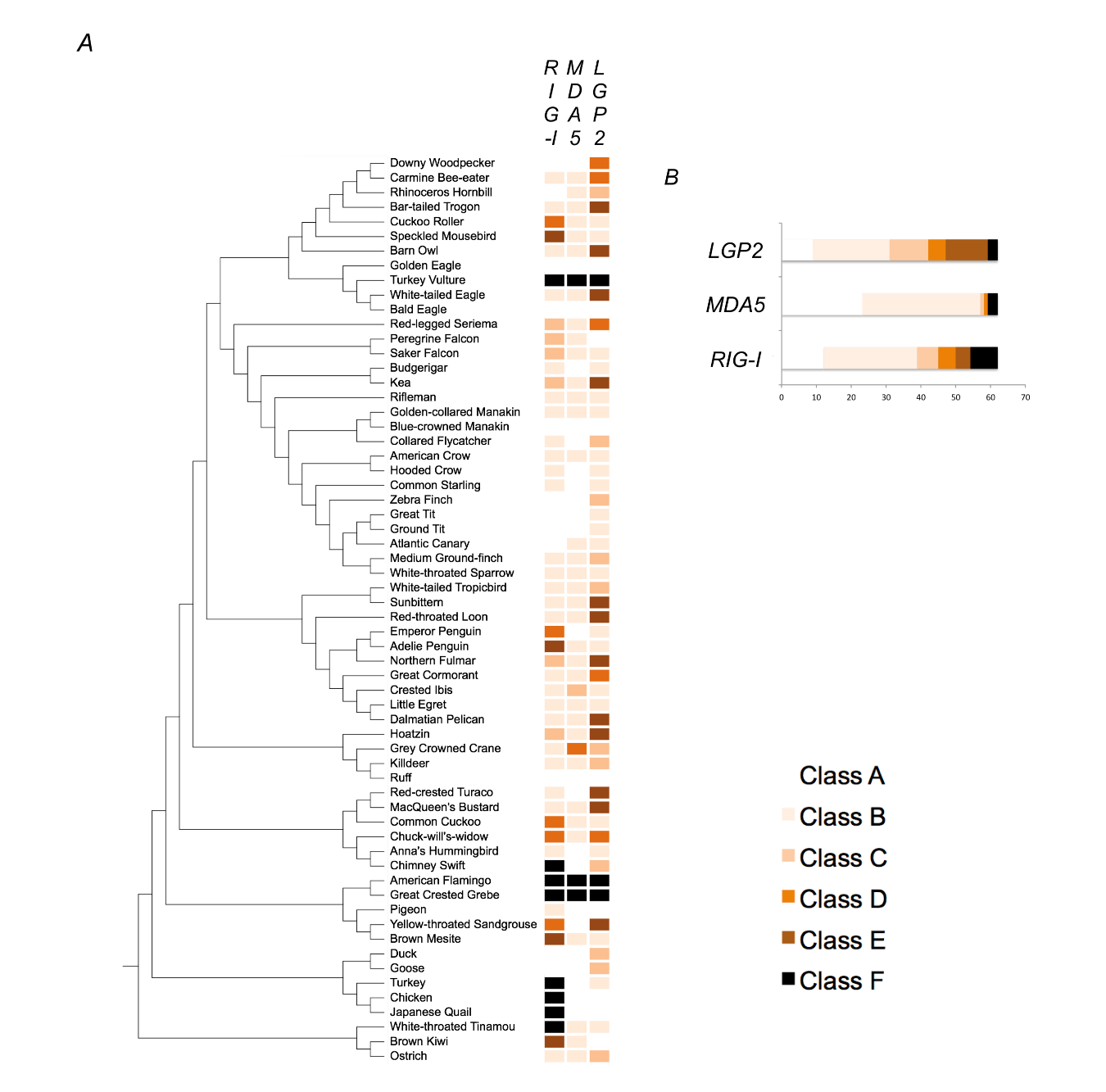


**Figure S1**. Six-class face quality assessment of the predicted avian RLR CDSs. The six colors of the blocks are indicative of the assigned classes as denoted in lower right corner of the figure. (A) Six-class assessment of the three RLR CDSs for each of the 62 species. The tree shows the phylogenetic relationships of the birds without being scaled on divergence time. (B) Summary of six-class assessment for each avian RLR.

**
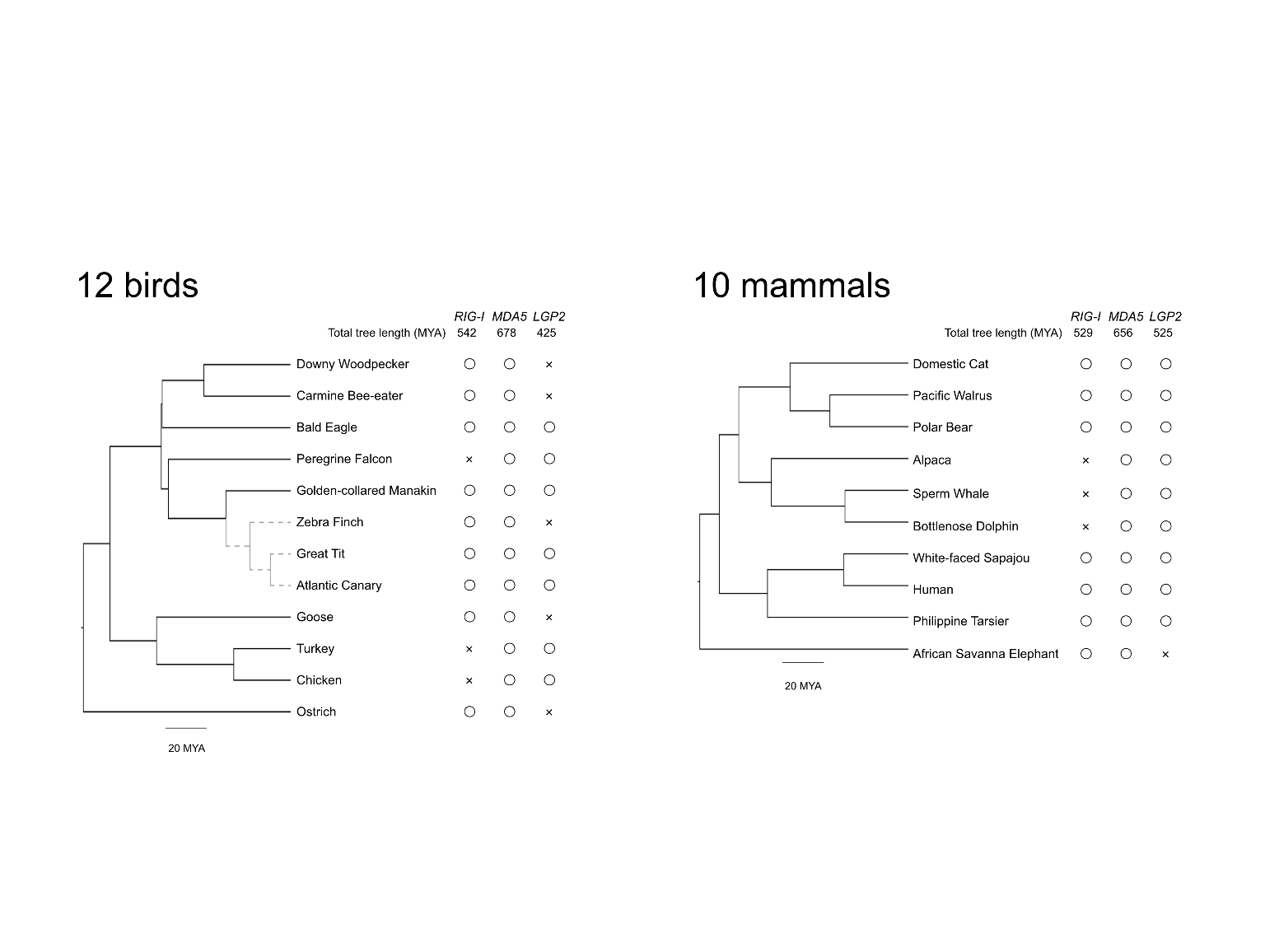
Figure S2.** Phylogeny of the species used for comparative analyses of RLRs between birds and mammals (dataset 3). The tree topology shows the phylogenetic relationship. A circle or a cross indicates the presence or absence, respectively, of a reliable CDS record from our dataset for birds or from GenBank for mammals. Branch lengths are in the scale of species divergence time. Branches in dotted line have an unknown divergence time and thus are not scaled. Total branch lengths denoted for each RLR gene are calculated with the species with a reliable CDS record of that gene. Total branch lengths involving branches of unknown divergence time take the average of possible maximum and minimum.


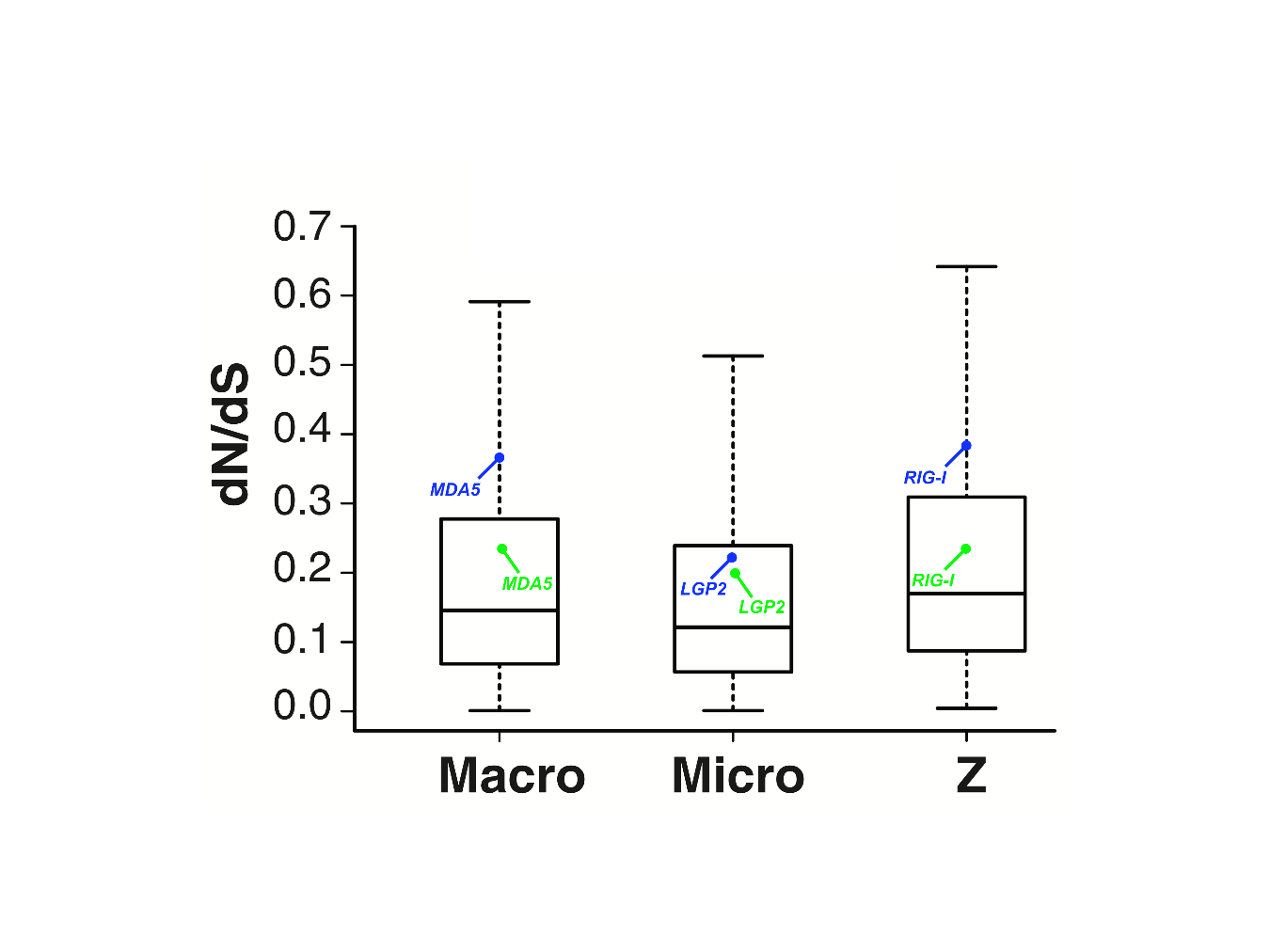


**Figure S3.** dN/dS of the three avian RLRs compared with average level of coding genes in birds. The figure is adapted from one in the literature (Zhang et al. 2014). Blue represents the result of SLAC using dataset 2 and green represents the result of Nei-Gojobori method using dataset 3.

Zhang et al. Comparative genomics reveals insights into avian genome evolution and adaptation. *Science* **2014**, *346*, 1311–1320, doi:10.1126/science.1251385.


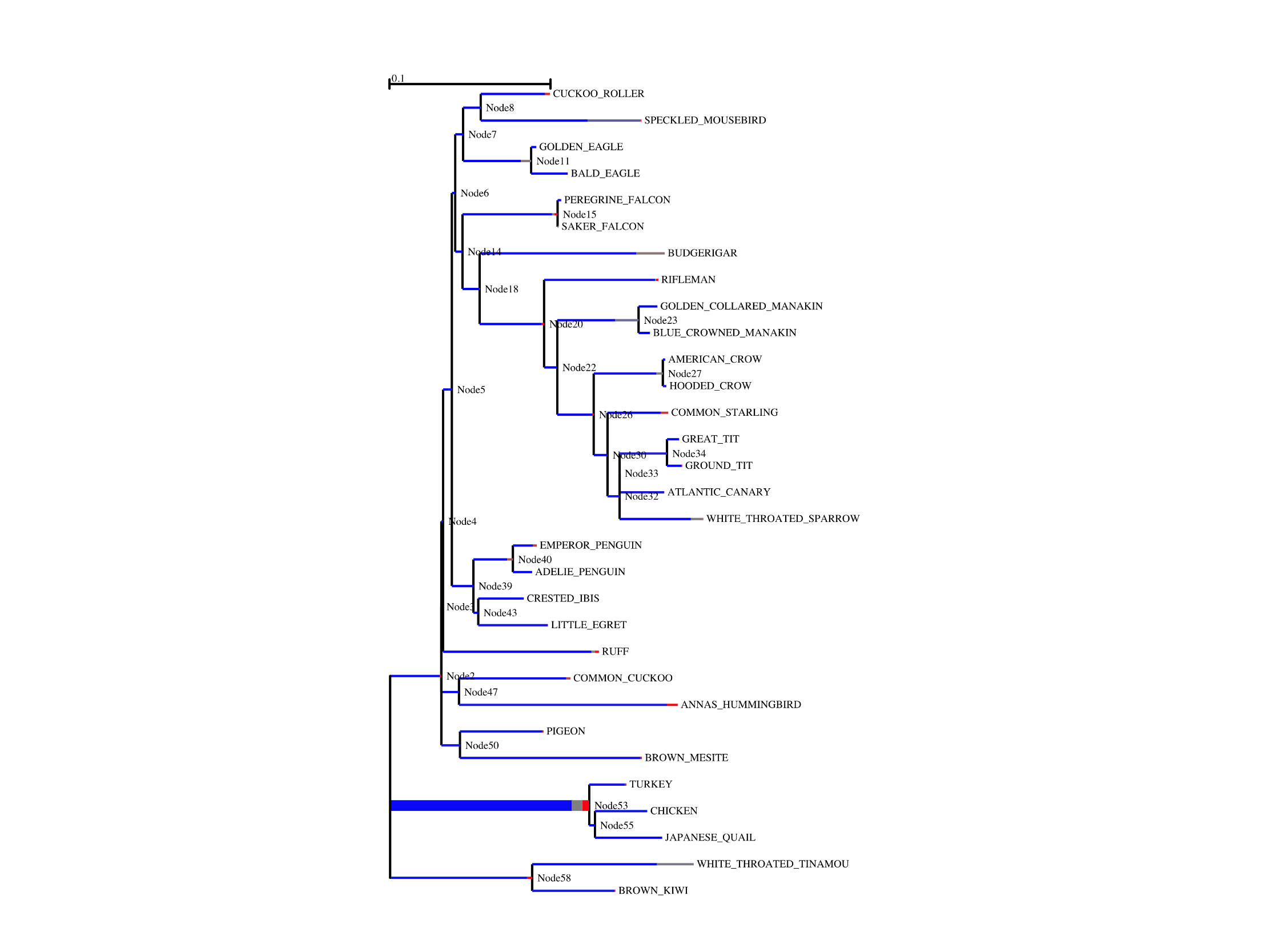


**Figure S4.** BSR analysis of avian LGP2. The tree topology shows the phylogenetic relationship of the birds. Strength of selection are represented by colors, with red corresponding to dN/dS > 5, blue to dN/dS = 0 and grey to dN/dS = 1. The width of each color component represents the proportion of sites in the corresponding class. Thicker branches have been classified as undergoing episodic positive selection by the sequential likelihood ratio test at corrected p ≤ 0.05.


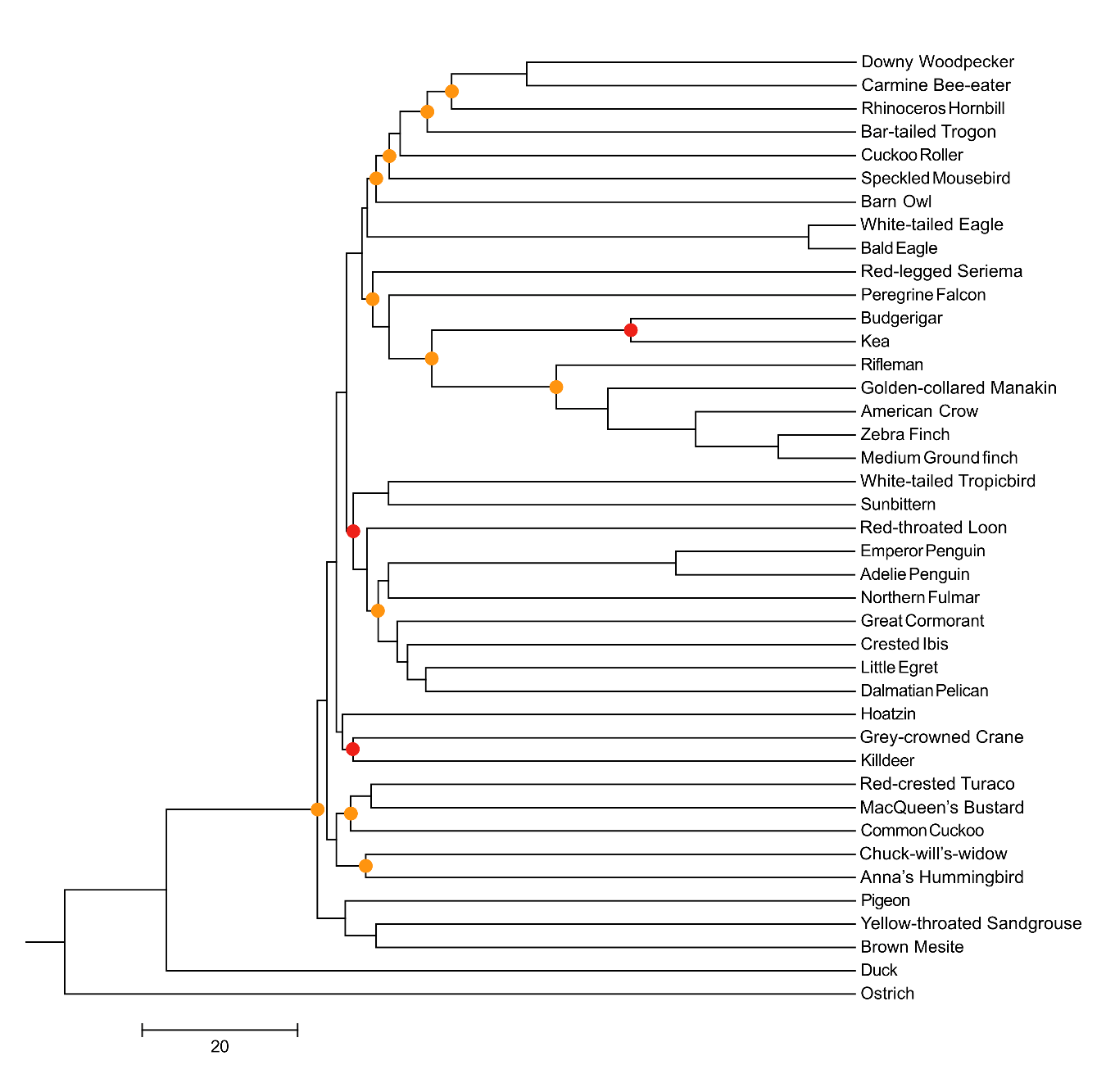


**Figure S5.** Nodes showing large discordance between their ranks of the PICs of species-to- ancestor dN/dS and ERV abundance. Dots in red indicate the nodes that have a difference of > 30 between the two ranks; in orange indicate the nodes that have a difference of > 20 but ≤ 30 between the two ranks. Branch lengths are scaled to divergence time (Mya).
